# *N*-Terminus Does Not Govern Protein Turnover of *Schizosaccharomyces pombe* CENP-A

**DOI:** 10.3390/ijms21176175

**Published:** 2020-08-26

**Authors:** Hwei Ling Tan, Yi Bing Zeng, Ee Sin Chen

**Affiliations:** 1 Department of Biochemistry, Yong Loo Lin School of Medicine, National University of Singapore, Singapore 117596, Singapore; hwei.ling.tan@u.nus.edu (H.L.T.); yibingzzz@u.nus.edu (Y.B.Z.); 2 National University Health System (NUHS), Singapore 119228, Singapore; 3 NUS Graduate School of Integrative Sciences & Engineering, National University of Singapore, Singapore 117456, Singapore

**Keywords:** CENP-A, centromere, chromosome segregation, fission yeast, *N*-terminus, protein turnover

## Abstract

Centromere integrity underlies an essential framework for precise chromosome segregation and epigenetic inheritance. Although centromeric DNA sequences vary among different organisms, all eukaryotic centromeres comprise a centromere-specific histone H3 variant, centromeric protein A (CENP-A), on which other centromeric proteins assemble into the kinetochore complex. This complex connects chromosomes to mitotic spindle microtubules to ensure accurate partitioning of the genome into daughter cells. Overexpression of CENP-A is associated with many cancers and is correlated with its mistargeting, forming extra-centromeric kinetochore structures. The mislocalization of CENP-A can be counteracted by proteolysis. The amino (*N*)-terminal domain (NTD) of CENP-A has been implicated in this regulation and shown to be dependent on the proline residues within this domain in *Saccharomyces cerevisiae* CENP-A, Cse4. We recently identified a proline-rich GRANT motif in the NTD of *Schizosaccharomyces pombe* CENP-A (SpCENP-A) that regulates the centromeric targeting of CENP-A via binding to the CENP-A chaperone Sim3. Here, we investigated whether the NTD is required to confer SpCENP-A turnover (i.e., counter stability) using various truncation mutants of SpCENP-A. We show that sequential truncation of the NTD did not improve the stability of the protein, indicating that the NTD of SpCENP-A does not drive turnover of the protein. Instead, we reproduced previous observations that heterochromatin integrity is important for SpCENP-A stability, and showed that this occurs in an NTD-independent manner. Cells bearing the null mutant of the histone H3 lysine 9 methyltransferase Clr4 (*Δclr4*), which have compromised constitutive heterochromatin integrity, showed reductions in the proportion of SpCENP-A in the chromatin-containing insoluble fraction of the cell extract, suggesting that heterochromatin may promote SpCENP-A chromatin incorporation. Thus, a disruption in heterochromatin may result in the delocalization of SpCENP-A from chromatin, thus exposing it to protein turnover. Taken together, we show that the NTD is not required to confer SpCENP-A protein turnover.

## 1. Introduction

Precise partitioning of chromosomes during mitosis is critical for genomic stability, and the loss of faithfulness during the segregation of genetic and epigenetic information is associated with many human diseases, including cancers and birth defects [1,2]. Chromosome segregation is brought about by the attachment of mitotic spindle microtubules that emanate from centrosomes (or spindle pole bodies) at cell poles to the kinetochores, which are specialized mega-protein complexes assembled onto chromosomal loci called centromeres [3,4]. The centromere consists of a higher order chromatin architecture that performs many critical mitotic functions, such as inculcating mechanical strength, to safeguard the structural integrity of the chromosomes; checkpoint functions, to ensure precise timing of chromosomal separation; and bi-directional orientation of the kinetochore-microtubule attachment [5,6,7].

Although the DNA sequence at the centromere is highly varied among eukaryotes, all functional centromeres, including neo-centromeres, host a centromere-specific histone H3 variant called centromeric protein A (CENP-A). CENP-A epigenetically marks the position of the centromere by assembling a specialized chromatin architecture, on which other centromere proteins of the constitutive centromere-associated network (CCAN) and the KNL-1/Mis12 complex/Ndc80 complex network (KMN) can be mounted for assembly of the kinetochore [6,8]. Overexpression of CENP-A has been reported in many types of cancer, including breast [9], lung [10], liver [11], bone [12], ovarian [13], and colorectal [14,15] cancers. Overexpressed CENP-A results in the formation of heterotypic nucleosomes, which can be ectopically mistargeted to non-centromeric loci [16]; these events are proposed to underlie the aneuploidy observed in cancer cells [14,17]. The mislocalization of overexpressed CENP-A to extra-centromeric loci has also been observed in fly and yeast [18,19,20] and observed to form ectopic kinetochores in fly [18]. The level of overexpressed CENP-A, along with that of other kinetochore proteins, may be used as a prognostic marker to predict patient survival and therapeutic response [21].

To restrict the localization of CENP-A to centromeres, the cell employs an E3 ubiquitin ligase through its association with the CENP-A-targeting domain (CATD) within the histone fold domain of CENP-A [22]. The E3 ligase then directs excessive CENP-A to the proteasome for degradation [22,23,24]. Several reports, however, also implicate the unstructured *N*-terminal domain (NTD) of yeast CENP-A in conferring ubiquitin-mediated proteolysis [19,25,26]; indeed, one study suggests that several proline residues nested in the NTD of *Saccharomyces cerevisiae* CENP-A (Cse4) are required for proline isomerase Fpr3-mediated proteolysis of Cse4 [27].

Recently, we interrogated the contribution of individual amino acid residues in the NTD of *Schizosaccharomyces pombe* CENP-A (SpCENP-A) [28]. We uncovered a proline-rich GRANT (Genomic stability-Regulating site within CENP-A *N*-Terminus) motif that undergoes *cis*–*trans* prolyl isomerization to regulate the association of the SpCENP-A NTD with the loading chaperone Sim3, which functions to target SpCENP-A to the centromere. In the present study, we investigated whether the proline-rich NTD of SpCENP-A also regulates SpCENP-A protein turnover and stability. We overexpressed several NTD-truncation mutants of SpCENP-A and tested the importance of NTD residues using a protein turnover assay [19,27]. We concluded that the short NTD of SpCENP-A is not required for protein stability but, instead, show that SpCENP-A turnover is observed in a manner independent of the NTD, and SpCENP-A stability is greatly attenuated in the absence of heterochromatin, as previously reported [29]. We propose that the NTD confers centromere targeting of SpCENP-A unstable proteins predominantly through a proteolysis-independent pathway.

## 2. Results

### 2.1. Creation of SpCENP-A N-Terminal Truncation (NT) Mutants

**Figure 1:**
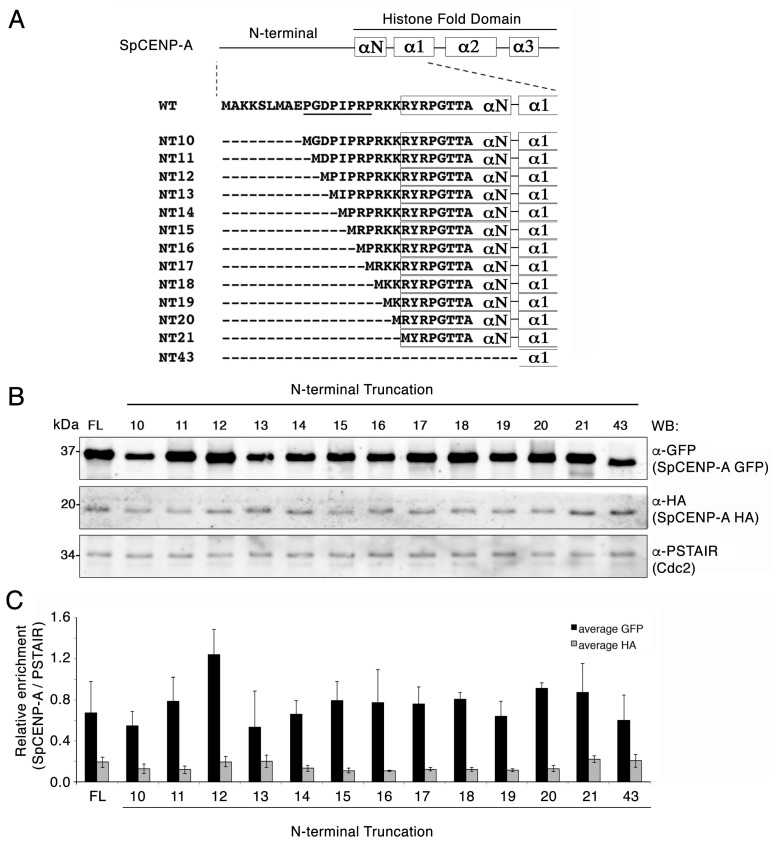
Systematic truncation of the amino acid residues in the *Schizosaccharomyces pombe* centromeric protein A (SpCENP-A) *N*-terminus. (**A**) Schematic diagram of SpCENP-A plasmid constructs with *N*-terminal truncations. NT, *N*-terminal truncation. Numbering indicates the amino acid residue truncation point relative to the first methionine. (**B**) Western blot examination of the SpCENP-A *N*-terminal mutant strains bearing a wild-type (WT) HA-tagged endogenous SpCENP-A gene, whereas the overexpressed mutant proteins from the *nmt41* promoter on a plasmid were GFP-tagged. Protein levels of SpCENP-A proteins were determined in cells after *nmt41* promoter induction by thiamine withdrawal from growth media. Similar levels of proteins were expressed among the exogenous SpCENP-A-GFP NTD (*N*-terminal domain) truncation mutants and the endogenous SpCENP-A-HA tagged protein. FL, full-length SpCENP-A protein; PSTAIR (Cdc2), loading control; HA, hemagglutinin, GFP, green fluorescent protein; kDa, kilodalton; WB, western blotting. (**C**) Quantification of the band intensities of the immunoblot shown in (**B**). Graph shows mean values of intensities. Bar, standard deviation. *p* > 0.05. Student’s *t*-test. Replicates from three independent experiments (average GFP or HA).

In our previous investigation into the role of the SpCENP-A NTD, we assessed the importance of the proline residues residing in the GRANT motif by overexpressing a mutant SpCENP-A^cnp1-4PA^ bearing alanine mutations at four proline residues (P10AP13AP15AP17A). We found that the SpCENP-A^cnp1-4PA^ mutant was almost as stable as the wild-type protein in a protein turnover assay [28]. These observations suggested that the ‘GRANT-proline’ residues have no effect on the proteasome-associated turnover of SpCENP-A protein. However, in our previous study, we did not address whether other residues in the SpCENP-A NTD may affect SpCENP-A protein turnover and stability. To systematically address this issue, we performed sequential truncation of the NTD of SpCENP-A, and expressed mutant proteins from an inducible *nmt41* promoter. These *N*-terminally truncated (NT) mutants exhibited progressively shorter *N*-termini, lacking the first 9 (NT10), 10 (NT11), 11 (NT12), 12 (NT13), 13 (NT14), 14 (NT15), 15 (NT16), 16 (NT17), 17 (NT18), 18 (NT18), 19 (NT20), 20 (NT21), and 42 (NT43) amino acid residues (Figure 1A). The NTD of SpCENP-A stretches from residue 1–20, hence NT21 represents complete deletion of the NTD. We also constructed NT43 containing a deletion of NTD, αN helix, and a portion of the α1 helix (Figure 1A).

The overexpressed GFP-tagged NT proteins were produced at an elevated level relative to the endogenous full-length protein (hemagglutinin (HA)-tagged) (Figure 1B,C). Truncation of the first 11 amino acids (NT12) appeared to slightly increase the steady state level of the expressed protein (albeit, not statistically significant), but further truncations did not affect the stabilization of the protein (Figure 1C).

### 2.2. Truncation of the NTD Does Not Destabilize the SpCENP-A Protein

Next, we assessed whether loss of the NTD—whole or in part—affects the stability of the SpCENP-A protein using measurements of protein turnover rate. Wild-type (WT) cells expressing the green fluorescent protein (GFP)-tagged full-length (FL) NT10, NT13, NT15, NT17, NT19, NT20, NT21, and NT43 SpCENP-A proteins from the *nmt41* promoter were incubated according to previously reported procedures [19,27]. Cells were incubated with 100 μg/mL cycloheximide (CHX) to block protein synthesis. Samples were collected at 30-min intervals for immunoblotting analysis. The level of α-tubulin was not affected by the addition of CHX over the course of the treatment (0–180 min) and was therefore used as a loading control.

**Figure 2:**
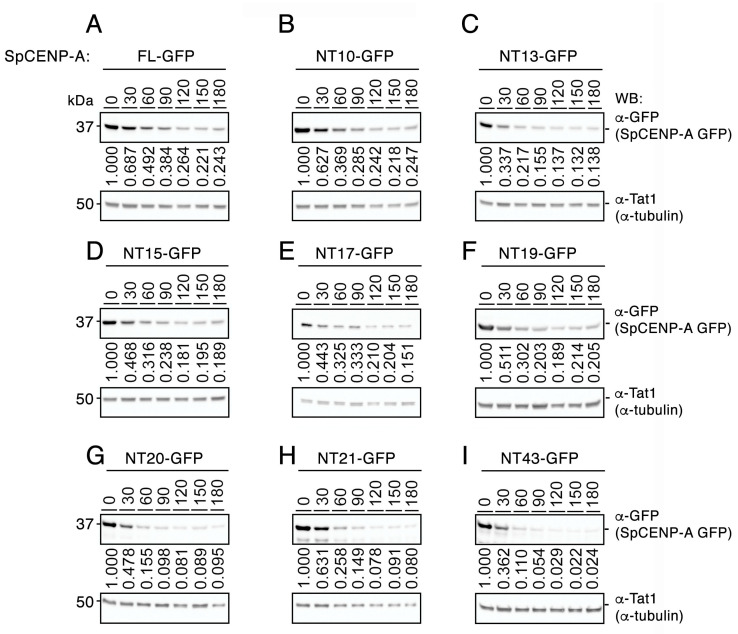
Truncation of the SpCENP-A NTD does not confer resistance to proteolytic degradation. Evaluation of exogenously expressed protein levels of GFP-tagged SpCENP-A *N*-terminal truncation mutants (NT-GFP) in a cycloheximide (CHX; 100 μg/mL) chase assay at 30 °C, 18 h post-induction after thiamine withdrawal. (**A**) FL-GFP, full-length SpCENP-A GFP-tagged protein, (**B**) NT10-GFP, (**C**) NT13-GFP, (**D**) NT15-GFP, (**E**) NT17-GFP, (**F**) NT-19-GFP, (**G**) NT20-GFP, (**H**) NT21-GFP, (**I**) NT43-GFP. Tat1 (α-tubulin), loading control; kDa, kilodalton; WB, western blotting. Relative protein enrichment from time 0 min (at 30 min intervals) to 180 min after exposure to CHX. Representative of two independent experiments.

Relative to α-tubulin, FL SpCENP-A-GFP appeared rather unstable, with a decreasing level of expression over the time course of the experiment (half-life: ~60 min; Figure 2A). With progressive truncation of the NTD, we did not observe any stabilization of the SpCENP-A protein (Figure 2B–H); instead, we observed an increase in protein turnover for all SpCENP-A mutant proteins, with an approximately two-fold reduced half-life (~30 min) relative to the FL protein. Additional truncation in the α-helical structure (αN and α1) led to a further increase in protein turnover, concomitant with an increased destabilization of the protein, and further shortening of the half-life to less than 30 min (Figure 2I).

To ascertain whether the bulky GFP tag affects the stability of the SpCENP-A protein, we fused a smaller FLAG-tag to SpCENP-A, and expressed the constructs in WT cells. Similar to the FL SpCENP-A-GFP (Figure 2A), as the protein level decreased, there was an increase in turnover of the FL SpCENP-A-FLAG proteins, and this was associated with increased length of CHX incubation (Figure S1). The stability of the FL SpCENP-A protein was comparable between the FLAG- and GFP-tagged constructs, with a similar half-life (~30 min). A residual level of protein, corresponding to about 20% of the total at time 0 min, persisted until the end of the experiment (180 min) for both tags. These results suggest that the epitope tag probably exerted little effect on the protein stability of FL SpCENP-A protein ectopically expressed from the REP41 plasmid.

**Figure 3:**
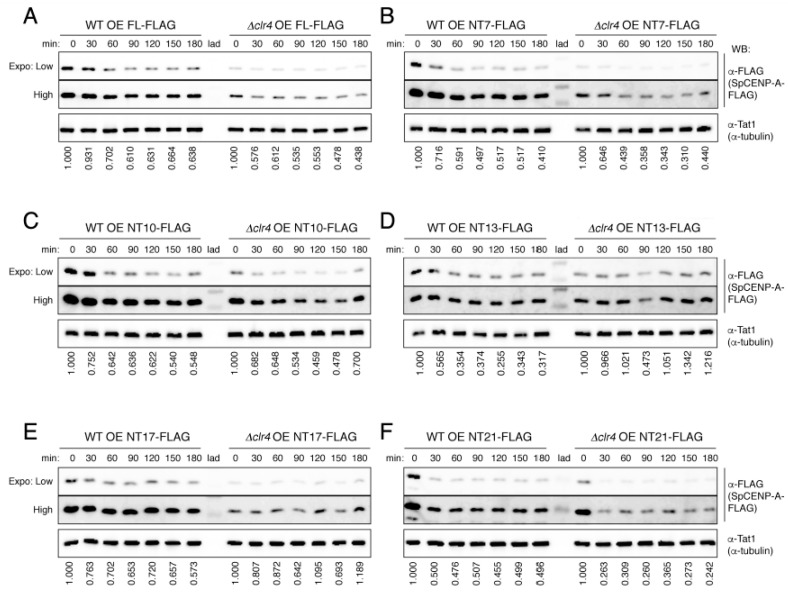
Heterochromatin-mediated protection of SpCENP-A against proteolytic degradation. Cycloheximide (CHX) chase assay of wild-type and heterochromatin-disrupted *Δclr4* cells expressing SpCENP-A FLAG-tagged *N*-terminal truncation (NT) mutants, 18 h post-induction after thiamine withdrawal. (**A**) FL-FLAG, full-length SpCENP-A FLAG-tagged protein, (**B**) NT7-FLAG, (**C**) NT10-FLAG, (**D**) NT13-FLAG, (**E**) NT17-FLAG, (**F**) NT21-FLAG. Tat1 (α-tubulin), loading control. Relative protein enrichment from time 0 min (at 30-min intervals) to 180 min after exposure to CHX. The intensity value of each band was normalized to that of 0 min of each strain. Representative of two independent experiments. OE, overexpression; lad, molecular weight ladder; WB, western blotting.

We next constructed the FLAG-tagged NT mutant proteins to confirm the findings in the protein stability assay (Figure 3). As with the GFP-tagged proteins, we observed no differences in the stabilization of the SpCENP-A proteins among any of the NTD mutants, confirming that the NTD of SpCENP-A is not required to regulate protein stability of SpCENP-A.

### 2.3. Heterochromatin Synergistically Acts with NTD to Safeguard the Integrity of SpCENP-A Protein

It was reported previously that heterochromatin is required to establish SpCENP-A chromatin at the fission yeast centromere [30,31], and that localization of SpCENP-A to centromeres is disrupted in cells expressing the null mutant of the histone H3 lysine 9 methyltransferase Clr4 (*Δclr4*) [29], which have compromised constitutive heterochromatin integrity [32,33]. Heterochromatin also protects SpCENP-A from ubiquitin-mediated degradation [29]. We therefore tested whether the absence of heterochromatin could increase the turnover of SpCENP-A with FL and partially truncated NTD by repeating the protein stability experiments in *Δclr4* cells (Figure 2). Protein stability was investigated for six constructs (FL, NT7, NT10, NT13, NT17, and NT21) relative to the respective levels of the WT (Figure 3A–F).

Consistent with previous reports [29], we observed a prominent decrease in the stability of SpCENP-A proteins in *Δclr4* cells. The protein level was so low that we had to saturate the intensity of the bands in the WT background (Figure 3). SpCENP-A was reduced at 0 min, almost to the level that remained in the cells across all constructs, including the FL and NT7-21 (Figure 3A–F). The levels of the FL and NT SpCENP-A FLAG (NT7, NT10, NT13, NT17, and NT21) constructs were reduced considerably to residual levels within 30–60 min, which persisted until the end of the experiment (Figure 3A–F); this was somewhat similar to the turnover pattern in the WT cells (Figure 2). Hence, it appeared that Clr4 affected a general process, possibly one involving the proper incorporation of the ectopically expressed SpCENP-A proteins into chromatin, such that excess levels of SpCENP-A in the soluble non-chromatin-bound fraction underwent proteolysis to prevent mislocalization to extra-centromeric loci [22,23,34].

### 2.4. Effect of Heterochromatin on SpCENP-A in Sub-Cellular Fractions

**Figure 4:**
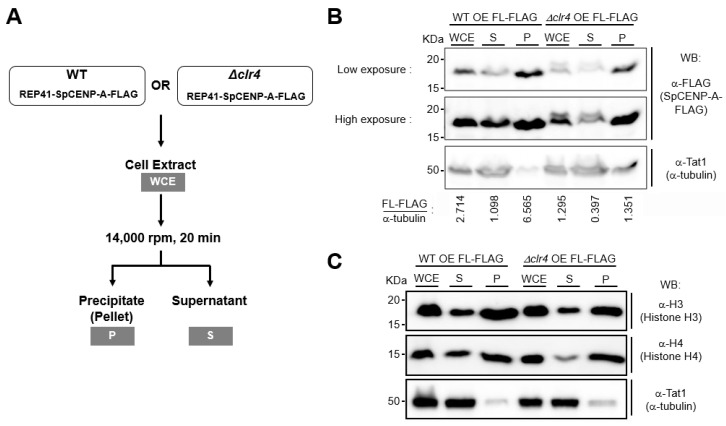
Levels of non-chromatin-bound (soluble) and chromatin-bound (insoluble) fractions in WT and *Δclr4* cells. (**A**) Schematic representation of steps for sub-cellular fractionation of cell extracts performed to separate the soluble and insoluble fractions. (**B**) Immunoblotting was performed to assess the level of full-length SpCENP-A in the whole cell extract (WCE) and soluble (S) non-chromatin-bound and insoluble (P, precipitate) fractions. α-Tubulin was used as a loading control. Relative fold enrichment in each fraction was calculated by normalizing the band intensities of SpCENP-A FLAG with that of α-tubulin. (**C**) The levels of histones H3 and H4 in the WCE, S, and P in the samples in (**B**). kDa, kilodalton; WB, western blot; OE, overexpression (using REP41).

To ascertain whether heterochromatin influences the proportion of SpCENP-A that is incorporated into chromatin, we performed a sub-cellular fractionation experiment by separating the soluble non-chromatin fraction from the insoluble fractions (i.e., those that contain chromatin) by centrifugal sedimentation (Figure 4A) [35] in WT and *Δclr4* cells. We observed that SpCENP-A mostly localized to the insoluble precipitated pellet in WT cells (P, Figure 4B, left), which was also enriched with canonical histones H3 and H4 (Figure 4C). The level of SpCENP-A in the whole cell extract (WCE) of *Δclr4* cells was reduced, as previously observed (Figure 3), as was the proportion of SpCENP-A in the insoluble fraction, compared to the WT control (P, Figure 4B, right). The levels of histones H3 and H4, however, did not seem to show a similar reduction in *Δclr4* relative to the WT control (Figure 4C). This suggests that chromatin-bound SpCENP-A is reduced in *Δclr4*, in which heterochromatin is compromised [32,33].

## 3. Discussion

In this study, we investigated the requirement of the SpCENP-A NTD in protein stability using full-length and sequentially truncated NTDs. We observed that partial or complete removal of the SpCENP-A NTD did not stabilize the protein, which differs from that observed for budding yeast Cse4. Yang and colleagues previously showed that heterochromatin protected overexpressed Cse4 in fission yeast from ubiquitin-dependent degradation in a manner that was dependent on the NTD of Cse4 [29]. Heterochromatic transcriptional silencing is enforced by the histone methyltransferase, Clr4, by catalyzing the methylation of histone H3 lysine 9. Therefore, we tested the contribution of Clr4 on SpCENP-A protein turnover using full-length or NTD truncation mutants. We observed that SpCENP-A proteins became highly unstable in the *clr4* deletion mutant, and this low stability was correlated with a decrease in chromatin-bound SpCENP-A. This suggests that heterochromatin somehow maintains SpCENP-A in the centromeric chromatin and, when heterochromatin is disrupted, SpCENP-A drops out from the chromatin into the soluble fraction, exposing it to degradation by the proteasome. Taken together, our results support the conclusion that the NTD of SpCENP-A does not contain the signal to confer the degradation of SpCENP-A, such that the protein would become stabilized in the absence of this signal.

CENP-A is an important epigenetic determinant of centromeric identity. The precise localization of CENP-A to the centromere is essential for governing the integrity of centromeric chromatin and genomic stability. The localization of CENP-A to centromeric regions is dependent on a CENP-A-targeting domain (CATD) nested within the histone fold domain of CENP-A, which interacts with the CENP-A loading factor Holliday Junction Recognition Protein (HJURP) and stabilizes CENP-A–histone H4 interaction [36,37]. Recently, it has been shown by us and others that the flexible NTD tail of CENP-A directs CENP-A centromeric targeting independently of the CATD via binding with CENP-A loading factors such as Sim3/Nuclear Autoantigen Sperm Protein (NASP) [28,38], the CCAN component CENP-T [39], and CENP-B [28,40]. We have further shown that the Sim3–SpCENP-A interaction is mediated via prolyl *cis*–*trans* isomerization of a proline-rich GRANT motif within the SpCENP-A NTD [28]. Proline residues regulate the stability of CENP-A^CSE4^ in budding yeast, and it has been suggested that the process also relies on proline isomerization; albeit, this is not supported by experimental evidence [27]. Loss of function of the NTD and the proline residues can stabilize CENP-A to encourage targeting to extra-centromeric regions, and may also result in the disruption of proper chromosome segregation. For this reason, it was important to determine whether chromosome mis-segregation is partly contributed by the perturbed stability of the SpCENP-A protein. We therefore investigated the contribution of the NTD in SpCENP-A turnover by studying the stability of the full-length and mutant proteins bearing NTD truncations. The absence of enhanced stabilization of SpCENP-A led us to conclude that the NTD does not regulate protein turnover of SpCENP-A. In fact, SpCENP-A becomes more unstable when the NTD is fully truncated and this instability is exacerbated when the αN helix is concomitantly shortened.

The mechanism that mediates the formation of the centromere on a particular chromosomal locus is unclear, but several studies in the fission yeast, *Neurospora crassa*, and human cells have shown that heterochromatin can direct CENP-A loading into juxtaposing centromeric sequences [31,41,42,43,44]. The exact molecular mechanism by which heterochromatin can direct the localization of CENP-A is still elusive, but heterochromatin could provide a favorable environment to attract important chromatin remodeling and histone-modifying factors [31]; for instance, via the displacement of the histone H2A.Z variant to permit the association of Scm3/HJURP [43]. An investigation of the *N*-terminal and *C*-terminal Cse4/Cnp1 chimera proteins in fission yeast showed that heterochromatin directs the centromeric localization of CENP-A by protecting it against ubiquitin-dependent degradation, which is mediated by the NTD of CENP-A [29]. Our previous work showed that the SpCENP-A NTD binds the Sim3 histone chaperone to determine SpCENP-A centromeric localization; however, our findings here do not support the view that the SpCENP-A NTD mediates protein degradation. Our data does support previous observations that heterochromatin is nevertheless required for SpCENP-A stability. Our sub-cellular fractionation assay showed an obvious reduction in chromatin-bound SpCENP-A in the absence of heterochromatin, supporting the hypothesis that heterochromatin may enforce an environment for SpCENP-A chromatin incorporation. It is possible that the augmented degradation of the SpCENP-A in the absence of heterochromatin (such as in the *clr4* null mutant) may be due to the increased dissociation of SpCENP-A and/or SpCENP-A-localizing factors into the non-chromatin fraction, thus exposing it to proteome-mediated degradation.

CENP-A is the epigenetic determinant that mediates inheritance of the centromeric position. To confer this function, it would be expected that CENP-A would be relatively stable. However, we were surprised that SpCENP-A protein turnover is relatively quick (half-life of 30 min), as similarly observed for budding yeast Cse4 [26,27]. It is possible that these proteins were ectopically expressed in an upregulated amount in the soluble non-chromatin-bound fraction, which exposes them to proteosomal degradation. Our sub-cellular fractionation results, however, do not support this possibility, as most of the overexpressed CENP-A was found in the insoluble fraction, suggesting chromatin incorporation (Figure 4B). A recent study reported the high turnover and constant replenishment of human CENP-A, even at the centromeres of quiescent cells, which do not undergo cell division [45]. The transcription of centromeric sequences is thought to act as a disruptive force that counters the stable association of CENP-A at the centromere, resulting in constant delocalization of CENP-A from the centromere [45,46]. CENP-A turnover at the centromere allows cells to adjust to the different levels of CENP-A, which may constitute an important developmental signal [45]. Hence, the rapid turnover of CENP-A could play an as yet unidentified physiological role.

Even though SpCENP-A undergoes constant turnover, we consistently observed a residual level of SpCENP-A that persisted throughout the course of the experiment for most of the constructs (FL and NT10–19) (Figure 2A–F). However, this residual level decreased somewhat when the entire NTD was removed (NT20, 21, and 43; Figure 2G–I). This result suggests that there may exist different populations of SpCENP-A that are differentially exposed to degradation, probably due to the higher-order compaction of the centromeric chromatin. It is possible that this residual population may represent the SpCENP-A nucleosome at the core of centromeric chromatin, and may represent the stable population that inherits the epigenetic information within the centromeric chromatin.

## 4. Materials and Methods

### 4.1. Schizosaccharomyces pombe Growth Media and Strains

We followed previously described protocols for handling the fission yeast, *Schizosaccharomyces pombe* [28,47]. The growth media used in this study, YEA and EMM-Leu, were prepared as described [48]. Strains used in this study are listed in Tables S1 and S2. Antibodies used in this study are listed in Table S3.

### 4.2. Contruction of SpCENP-A N-Terminal Truncation Plasmids and Escherichia coli Cell Transformation

Genomic DNA from wild-type fission yeast cells was used as a template for the PCR amplification of *N*-terminally truncated CENP-A gene copies using Phusion polymerase (Thermo Fisher Scientific, Waltham, MA, USA), whereas plasmids pFA6a-6xGLY-3xFLAG-kanMX6 [49] and pFA6a-GFP(S65T)-kanMX6 [50] were used as templates [50] for FLAG and GFP protein tags, respectively. All PCR products were ethanol-precipitated and subjected to gel purification using a DNA gel extraction kit (Qiagen) according to manufacturer’s instructions. Both plasmid pREP41 and purified DNA (PCR products) were treated with restriction enzymes (New England Biolabs (NEB), Ipswich, MA, USA) and subjected to agarose gel purification, as described previously [28]. Digested gene inserts were ligated to pREP41 plasmids with T4 ligase (NEB), and transformed into TOP10 competent cells (Thermo-Fisher Scientific), before plating onto LB + Car agar plates (Luria–Bertani medium with 100 μg/mL carbenicillin (GoldBio, St. Louis, MO, USA). Positive transformants were then expanded by plasmid miniprep (Qiagen, Hilden, Germany) and extracted plasmids were sequenced using the Sanger method for confirmation of the correct gene sequences, frame, and orientation.

### 4.3. Construction of Exogenously Expressed SpCENP-A NT Mutant Strains

Sequence-verified pREP41-SpCENP-A NT mutant plasmid constructs were transformed into strains with a *leu* nutrient selection marker, as described [48,50], using strains listed in Table S1. In brief, 20 mL of fresh log-phase fission yeast cells, prepared in an overnight culture to OD_600_ = 0.5, was used per transformation. The cells were pelleted at 5000× *g*, 4 °C for 1 min, washed once in sterile water, then once in LiOAc-TE (0.1 M lithium acetate (pH 7.4), 10 mM Tris-HCl (pH 7.5), and 1 mM EDTA), and then resuspended to 1/200th of the original culture volume in LiOAC-TE. For each plasmid DNA transformation, 100 μL of cell suspension was used. To this cell suspension, we added 3 μL of each plasmid DNA mixed with 2 μL of carrier DNA (Salmon testes DNA; Sigma-Aldrich, St Louis, MO, USA) and 260 μL of PEG-LiOAC-TE (40% (*v*/*v*) PEG 4000, 0.1 M lithium acetate (pH 7.4), 10 mM Tris-HCl (pH 7.5), and 1 mM EDTA). The cell suspension was then incubated at 26–30 °C for 1 h. Before heat shock, 43 μL DMSO was added to the cell suspension. Heat shock was performed at 42 °C for 5 min, followed by a 5 min rest phase at room temperature. The transformed cells were then pelleted, washed once in sterile water, and plated on the selective growth media. The expression of the transformed plasmids or epitope-fused genes was confirmed by immunoblotting.

### 4.4. Cycloheximide Chase Assay

SpCENP-A NT mutant overexpression strains were pre-inoculated and incubated in EMM-Leu media supplemented with 100 μM of thiamine overnight at 30 °C with shaking. The induction of exogenous protein expression in log-phase strains bearing CENP-A *N*-terminal truncation plasmid constructs was performed by thiamine withdrawal for 18 h at 30 °C, as previously described [28], or at 33 °C. The evaluation of protein stability was performed by the addition of 100 μg/mL cycloheximide (final concentration) (Sigma-Aldrich) to the induced cell cultures. Cell cultures (10 mL) were collected and pelleted upon the addition of cycloheximide, and thereafter at 30-min intervals until 180 min [27]. Total cell proteins were extracted by trichloroacetic acid (TCA; Sigma-Aldrich) and SpCENP-A protein levels were evaluated by western blotting and immunostaining according to previously published procedures [28,51], with antibodies employed as listed in Table S3.

### 4.5. Sub-Cellular Fractionation

Full-length SpCENP-A was overexpressed in log-phase WT or *Δclr4* strains in the absence of thiamine repression for 18 h at 30 °C. The cells were then harvested and lysed in 1× HB buffer as previously described [28]. The whole cell extracts were centrifuged at 14,000 rpm, 20 min at 4 °C. Equal volumes of 1× Laemmli buffer were added to each of the seperated pellet (*v*/*v*) and supernatant (*v*/*v*) and heat-denatured. The solubilized proteins were then assessed by western blotting.

### 4.6. Statistical Tests for Significance

Statistical significance, denoted by *p* values less than 0.05, was determined using the Student’s *t*-test. Statistical analyses and graphical output were created using Microsoft Office Excel.

## 5. Conclusions

In conclusion, our work showed that the SpCENP-A NTD does not regulate protein stability of SpCENP-A, which, however, is dependent on heterochromatin integrity in an NTD-independent manner. Heterochromatin may enhance protein stability of SpCENP-A via enforcing its chromatin incorporation.

## Supplementary Materials

Supplementary materials can be found at https://www.mdpi.com/1422-0067/21/17/6175/s1. Supplementary materials include three tables (Tables S1, S2 and S3) and one figure (Figure S1). Table S1: Genotype of integrant strains used in this study; Table S2: Genotype of overexpression strains and plasmid constructs synthesised for this study; Table S3: Antibodies used in study; Figure S1: Protein turnover of FLAG-tagged SpCENP-A under cycloheximide treatment.

## Abbreviations

CCAN	Constitutive centromere-associated network (of proteins)
CENP-A	Centromere protein A
CHX	Cycloheximide
clr4	Cryptic Loci Regulator 4 (histone lysine H3 methyltransferase Clr4)
Cnp1	CENP-A in *Schizosaccharomyces pombe*
Cse4	CENP-A in *Saccharomyces cerevisiae*
DMSO	Dimethyl sulfoxide
E3	Ubiquitin ligase E3
EMM (-leu)	Edinburgh minimal media (without leucine)
FL	Full-length
FLAG	FLAG (tag)
GFP	Green fluorescence protein (tag)
GRANT	Genomic stability-Regulating site within CENP-A *N*-Terminus motif
H3	Histone H3
HA	Hemagglutinin (tag)
kan	Kanamycin
KMN	KNL-1/Mis12 complex/Ndc80 complex
LB	Lysogeny broth, Luria–Bertani medium
leu	Leucine
max	Maximum
min	Minimum
*N*-terminal	NH_2_ (amino) terminal
NH_2_	Amino
NT	NH_2_-terminal truncation
NTD	NH_2_ (amino)-terminal domain
OE	Overexpression
PCR	Polymerase chain reaction
PEG-LiOAC-TE	Polyethylene glycol 4000-Lithium acetate-Tris-ethylenediaminetetraacetic acid
*S. pombe*	*Schizosaccharomyces pombe* (fission yeast)
*S. cerevisiae*	*Saccharomyces cerevisiae*
Sim3	Silencing in the Middle of the centromere 3 (NASP family CENP-A chaperone)
SpCENP-A	CENP-A in *Schizosaccharomyces pombe*
Tat1	α-Tubulin
TCA	Trichloroacetic acid
TE	Tris-EDTA buffer
WB	Western blot
WT	Wild-type
YEA	Yeast extract adenine
