# Supplementary material for: N-Terminus Does Not Govern Protein Turnover of Schizosaccharomyces pombe CENP-A
Source: Int J Mol Sci. 2020 Aug 26;21(17):6175. doi: 10.3390/ijms21176175 (PMC7503380; doi:10.3390/ijms21176175)
Supplement: Supplementary file 1 [file ijms-21-06175-s001.pdf]

International Journal of
Molecular Sciences

MDPI

Supplementary Materials

Table S1. Integrant strains used in this study.

Strain Name	Tagged Gene Locus	Tag/Deletion	Genotype	Source
HF123	-	-	Wild type, *h+*, *leu⁻*	*S. pombe* library
**972**	-	-	Wild type, *h+*, *lys⁺*	*S. pombe* library
**Ura-Cnp1HA**	cnp1^{ori} replacement, lys1^{ori} replacement	*ura4⁺* Cnp1-HA	*cnp1^{ori}::ura4^{+}, lys1^{+}::cnp1^{+}-HA, leu^{-}, h+*	This study
Δclr4	clr4^{ori} replacement	*kan⁺*	*clr4::kan^{+}, leu^{-}, h+*	*S. pombe* library

Table S2. Overexpression strains and plasmid constructs used in this study.

Strain Name	Host	Plasmid	Genotype	This study
**Vector**	HF123	*pREP41*	*h+, leu⁺*	This study
**WT/FL**	HF123	*pREP41-FL*	*h+, leu⁺*	This study
**NT9**	HF123	*pREP41-NT9*	*h+, leu⁺*	This study
**NT10**	HF123	*pREP41-NT10*	*h+, leu⁺*	This study
**NT11**	HF123	*pREP41-NT11*	*h+, leu⁺*	This study
**NT12**	HF123	*pREP41-NT12*	*h+, leu⁺*	This study
**NT13**	HF123	*pREP41-NT13*	*h+, leu⁺*	This study
**NT14**	HF123	*pREP41-NT14*	*h+, leu⁺*	This study
**NT15**	HF123	*pREP41-NT15*	*h+, leu⁺*	This study
**NT16**	HF123	*pREP41-NT16*	*h+, leu⁺*	This study
**NT17**	HF123	*pREP41-NT17*	*h+, leu⁺*	This study
**NT18**	HF123	*pREP41-NT18*	*h+, leu⁺*	This study
**NT19**	HF123	*pREP41-NT19*	*h+, leu⁺*	This study
**NT20**	HF123	*pREP41-NT20*	*h+, leu⁺*	This study
**NT21**	HF123	*pREP41-NT21*	*h+, leu⁺*	This study
**NT43**	HF123	*pREP41-NT43*	*h+, leu⁺*	This study
**FL-FLAG**	HF123	*pREP41-FL-FLAG*	*h+, leu⁺*	This study
**NT7-FLAG**	HF123	*pREP41-NT7-FLAG*	*h+, leu⁺*	This study
**NT9-FLAG**	HF123	*pREP41-NT9-FLAG*	*h+, leu⁺*	This study
**NT10-FLAG**	HF123	*pREP41-NT10-FLAG*	*h+, leu⁺*	This study
**NT11-FLAG**	HF123	*pREP41-NT11-FLAG*	*h+, leu⁺*	This study
**NT12-FLAG**	HF123	*pREP41-NT12-FLAG*	*h+, leu⁺*	This study
**NT13-FLAG**	HF123	*pREP41-NT13-FLAG*	*h+, leu⁺*	This study
**NT14-FLAG**	HF123	*pREP41-NT14-FLAG*	*h+, leu⁺*	This study
**NT15-FLAG**	HF123	*pREP41-NT15-FLAG*	*h+, leu⁺*	This study
**NT16-FLAG**	HF123	*pREP41-NT16-FLAG*	*h+, leu⁺*	This study
**NT17-FLAG**	HF123	*pREP41-NT17-FLAG*	*h+, leu⁺*	This study
**NT18-FLAG**	HF123	*pREP41-NT18-FLAG*	*h+, leu⁺*	This study
**NT19-FLAG**	HF123	*pREP41-NT19-FLAG*	*h+, leu⁺*	This study
**NT20-FLAG**	HF123	*pREP41-NT20-FLAG*	*h+, leu⁺*	This study
**NT21-FLAG**	HF123	*pREP41-NT21-FLAG*	*h+, leu⁺*	This study
**NT43-FLAG**	HF123	*pREP41-NT43-FLAG*	*h+, leu⁺*	This study
**Vector**	Ura-Cnp1HA	*pREP41*	*h+, leu⁺*	This study
**FL-GFP**	Ura-Cnp1HA	*pREP41-FL-GFP*	*h+, leu⁺*	This study
**NT10-GFP**	Ura-Cnp1HA	*pREP41-NT10-GFP*	*h+, leu⁺*	This study
**NT11-GFP**	Ura-Cnp1HA	*pREP41-NT11-GFP*	*h+, leu⁺*	This study
**NT12-GFP**	Ura-Cnp1HA	*pREP41-NT12-GFP*	*h+, leu⁺*	This study
**NT13-GFP**	Ura-Cnp1HA	*pREP41-NT13-GFP*	*h+, leu⁺*	This study
**NT14-GFP**	Ura-Cnp1HA	*pREP41-NT14-GFP*	*h+, leu⁺*	This study
**NT15-GFP**	Ura-Cnp1HA	*pREP41-NT15-GFP*	*h+, leu⁺*	This study
**NT16-GFP**	Ura-Cnp1HA	*pREP41-NT16-GFP*	*h+, leu⁺*	This study
**NT17-GFP**	Ura-Cnp1HA	*pREP41-NT17-GFP*	*h+, leu⁺*	This study
**NT18-GFP**	Ura-Cnp1HA	*pREP41-NT18-GFP*	*h+, leu⁺*	This study
**NT19-GFP**	Ura-Cnp1HA	*pREP41-NT19-GFP*	*h+, leu⁺*	This study
**NT20-GFP**	Ura-Cnp1HA	*pREP41-NT20-GFP*	*h+, leu⁺*	This study
**NT21-GFP**	Ura-Cnp1HA	*pRep41-NT21-GFP*	*h+, leu⁺*	This study
**NT43-GFP**	Ura-Cnp1HA	*pREP41-NT43-GFP*	*h+, leu⁺*	This study
**Vector**	*Δclr4*	*pREP41*	*h+, leu⁺*	This study
**FL-FLAG**	*Δclr4*	*pREP41-FL-FLAG*	*h+, leu⁺*	This study
**NT7-FLAG**	*Δclr4*	*pREP41-NT7-FLAG*	*h+, leu⁺*	This study
**NT9-FLAG**	*Δclr4*	*pREP41-NT9-FLAG*	*h+, leu⁺*	This study
**NT10-FLAG**	*Δclr4*	*pREP41-NT10-FLAG*	*h+, leu⁺*	This study
**NT11-FLAG**	*Δclr4*	*pREP41-NT11-FLAG*	*h+, leu⁺*	This study

**NT13-FLAG**	*Δclr4*	*pREP41-NT13-FLAG*	*h+, leu$^+$*	This study
**NT15-FLAG**	*Δclr4*	*pREP41-NT15-FLAG*	*h+, leu$^+$*	This study
**NT17-FLAG**	*Δclr4*	*pREP41-NT17-FLAG*	*h+, leu$^+$*	This study
**NT19-FLAG**	*Δclr4*	*pREP41-NT19-FLAG*	*h+, leu$^+$*	This study
**NT21-FLAG**	*Δclr4*	*pREP41-NT21-FLAG*	*h+, leu$^+$*	This study

Table S3. Antibodies used in study.

Antibody	Dilution	Origin	Manufacturer	Cat. No
Anti-FLAG	1:500	Mouse monoclonal	Wako	018-22381
Anti-GFP	1:500	Mouse monoclonal	Roche	11814460001
Anti-HA	1:500	Mouse monoclonal, clone: 12CA5	Roche	11666606001
PSTAIR (cdc2, p34)	1:500	Rabbit polyclonal	Santa cruz	SC-53
Anti-α Tubulin (αTat 1)	1:500	Mouse polyclonal	Gift from Keith Gull.	-
Anti-mouse IgG HRP	1:5000	Goat polyclonal	Santa cruz	SC-2004
Anti-rabbit IgG HRP	1:5000	Goat polyclonal	Santa cruz	SC-2005

Supplementary Figure S1: Protein turnover of FLAG-tagged SpCENP-A under cycloheximide treatment.

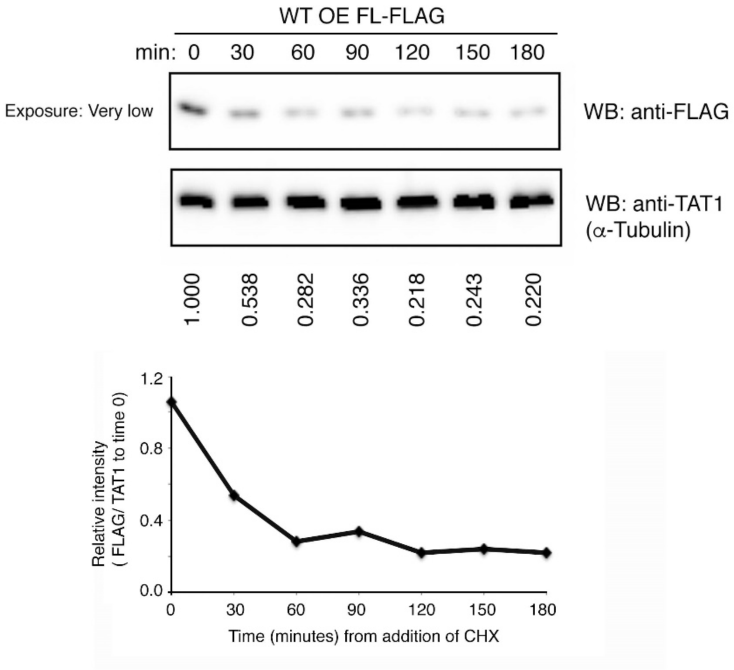

Figure S1. Very low exposure blot of cycloheximide chase assay of wild type strain *HF123* expressing Full length SpCENP-A-FLAG tagged, 30 °C, 18 h post induction in absence of thiamine. FL-FLAG; full length SpCENP-A FLAG tagged protein, CHX; cycloheximide, Tat1; α-tubulin; loading control. Relative protein enrichment to time 0 min (at 30 min intervals) to 180 min exposure to cycloheximide. Representative of two independent experiments. OE: overexpression, exposure: very low, WB: western blotting.

© 2020 by the author. Licensee MDPI, Basel, Switzerland. This article is an open access article distributed under the terms and conditions of the Creative Commons Attribution (CC BY) license (http://creativecommons.org/licenses/by/4.0/).
